# Parathyroid hormone change after cinacalcet initiation and one-year clinical outcome risk: a retrospective cohort study

**DOI:** 10.1186/s12882-015-0030-8

**Published:** 2015-03-31

**Authors:** Wendy L St. Peter, Akeem A Yusuf, Thy Do, Kimberly A Lowe, Jiannong Liu, Kimberly M Nieman, Brian D Bradbury, Allan J Collins

**Affiliations:** Chronic Disease Research Group, Minneapolis Medical Research Foundation, Minneapolis, Minnesota; University of Minnesota, College of Pharmacy, Minneapolis, Minnesota; Center for Observational Research, Amgen Inc., Thousand Oaks, California

**Keywords:** Cinacalcet, Hemodialysis, Parathyroid hormone, Secondary hyperparathyroidism

## Abstract

**Background:**

Cinacalcet reduces parathyroid hormone (PTH) levels in patients receiving hemodialysis, but no non-experimental studies have evaluated the association between changes in PTH levels following cinacalcet initiation and clinical outcomes. We assessed whether short-term change in PTH levels after first cinacalcet prescription could serve as a surrogate marker for improvements in longer-term clinical outcomes.

**Methods:**

United States Renal Data System data were linked with data from a large dialysis organization. We created a point prevalent cohort of adult hemodialysis patients with Medicare as primary payer who initiated cinacalcet November 1, 2004-February 1, 2007, and were on cinacalcet for ≥ 40 days. We grouped patients into quartiles of PTH change after first cinacalcet prescription. We used Cox proportional hazard modeling to evaluate associations between short-term PTH change and time to first composite event (hospitalization for cardiovascular events or mortality) within 1 year. Overall models and models stratified by baseline PTH levels were adjusted for several patient-related factors.

**Results:**

For 2485 of 3467 included patients (72%), PTH levels decreased after first cinacalcet prescription; for 982 (28%), levels increased or were unchanged. Several characteristics differed between PTH change groups, including age and mineral-and-bone-disorder laboratory values. In adjusted models, we did not identify an association between greater short-term PTH reduction and lower composite event rates within 1 year, overall or in models stratified by baseline PTH levels.

**Conclusions:**

Short-term change in PTH levels after first cinacalcet prescription does not appear to be a useful surrogate for longer-term improvements in cardiovascular or survival risk.

**Electronic supplementary material:**

The online version of this article (doi:10.1186/s12882-015-0030-8) contains supplementary material, which is available to authorized users.

## Background

Secondary hyperparathyroidism (SHPT) is managed primarily via clinical control of the biochemical parameters parathyroid hormone (PTH), calcium, and phosphorus. Cinacalcet (Sensipar®) has been shown to lower levels of all three of these, on average, in randomized clinical trials and observational studies [1-4]. Separately, cross-sectional and time-dependent values of PTH, calcium, and phosphorus, or mineral and bone disorder (MBD) phenotypes, have been associated with clinical outcomes in observational studies [5-8]. A recent cross-sectional study [7] using Dialysis Outcomes and Practice Patterns (DOPPS) data showed that PTH levels have risen in all included countries (except Japan) since 1996-2001, and that higher categories of PTH (>300 pg/mL) were associated with higher cardiovascular and all-cause mortality than the referent PTH category 150-300 pg/mL. Kalantar-Zadeh et al. showed that higher PTH levels were associated with increased mortality risk in adjusted time-dependent models [6]. We are not aware of any studies that looked directly at the association of PTH change and clinical outcomes after a specific therapy has been initiated. No studies have evaluated the association between short-term changes in PTH following first cinacalcet prescription and whether these early changes relate to clinical outcomes.

The implications of early change and associated outcomes may affect how cinacalcet is used under the proposed expansion of the US dialysis Prospective Payment System (PPS), or “bundle”. In 2024, it is anticipated that all chronic kidney disease MBD medications will be provided by dialysis units and reimbursed through the monthly dialysis PPS bundled payment, as erythropoiesis-stimulating agents, vitamin D, and iron products currently are [9]. In a bundled environment, it would be useful to know whether PTH change following cinacalcet prescription over a relatively short time period is associated with important outcomes such as mortality or cardiovascular events. In this study, we assessed whether short-term change in PTH levels after first cinacalcet prescription could be used as a surrogate for longer-term improvements in clinical outcomes.

## Methods

United States Renal Data System (USRDS) data were linked with data from a large dialysis organization by the USRDS. Laboratory and prescription data were obtained from the electronic medical records of the large dialysis organization. Variables included laboratory values (PTH, calcium, phosphorus, albumin, hemoglobin), weight and height to calculate body mass index (BMI), vascular access, dialysis dose (Kt/V), intravenous (IV) vitamin D use, and phosphate binder use. The USRDS database supplied information on patient demographics, primary ESRD cause, dialysis duration, comorbid conditions, hospitalizations, and mortality. We have previously described details regarding database linkage, variables, and drug file creation [4,10].

We selected our study population from a point prevalent cohort of 45,589 hemodialysis patients alive on August 1, 2004. Of these, we included adult (age ≥ 18 years) patients with Medicare as primary payer who were alive on November 1, 2004. We excluded patients who died, moved from the network of the large dialysis organization, changed modality (to peritoneal dialysis), underwent kidney transplant, or received cinacalcet before November 1, 2004. Included patients were those who received a first prescription for 30 mg of cinacalcet between November 1, 2004, and February 1, 2007, received intravenous IV vitamin D during the 3-month period before cinacalcet initiation, and had records of cinacalcet prescription covering at least 40 consecutive days after the first prescription date. We chose a cinacalcet dose of 30 mg because almost all patients are initiated at 30 mg and we could thus assess PTH change following a standard dose. In addition, we required patients to have a mean baseline PTH level of 300 pg/mL or higher (calculated as the average of all PTH measurements in the 90 days before cinacalcet initiation), and we required the most proximal pre-initiation PTH level to be ≥ 300 pg/mL to assure an indication for cinacalcet treatment.

We defined pre-initiation PTH as the value most proximal to first cinacalcet prescription. Cinacalcet steady-state blood levels are achieved by day 7 of daily dosing, and PTH levels appear to stabilize in that timeframe with the same daily dose [11,12]. We determined from preliminary analysis that most patients initiating cinacalcet have a PTH level drawn within 40 days of initiation. Thus, we defined post-initiation PTH as the value between days 8 and 40 after first cinacalcet prescription; if more than one PTH level was drawn during that timeframe, we chose the one closest to day 40 to allow patients time to establish daily dosing patterns. The percentage change in PTH was defined as the change between pre-initiation and post-initiation levels.

We grouped included patients based on quartiles of PTH decrease from first cinacalcet prescription. Patients whose PTH levels increased or remained unchanged were grouped together separately. In a Phase 2 study, all patients receiving doses of 25 mg or higher exhibited a decrease in PTH level following cinacalcet treatment; hence, lack of a decrease might represent treatment non-adherence [12].

We evaluated patient characteristics at baseline for each group: age, race, sex, cause of ESRD, dialysis duration, BMI, SHPT-related laboratory values (PTH, pg/mL; calcium, mg/dL; phosphorus, mg/dL), Kt/V, hospital days, phosphate binder use at the time of first cinacalcet prescription, and 11 comorbid conditions (atherosclerotic heart disease [ASHD], congestive heart failure [CHF], cerebrovascular accident/transient ischemic attack, peripheral vascular disease [PVD], other cardiac disease, chronic obstructive pulmonary disease, gastrointestinal disease, liver disease, dysrhythmia, cancer, and diabetes). A comorbid condition was considered present if one inpatient or two outpatient diagnosis codes for the condition appeared during the baseline 6 months [13]. Age and dialysis duration were determined at date of cinacalcet initiation. BMI was the average over the 30 days before cinacalcet initiation. Calcium, phosphorus, and Kt/V were calculated as mean values over the 3-month baseline period before cinacalcet initiation, and phosphate binder use was evaluated during this same period.

Our primary outcome of interest was a composite of hospitalization for acute myocardial infarction (AMI), stroke, CHF, or death. We required that the following primary diagnosis codes be present for hospitalization events: 410.x0, 410.x1 for AMI; 430, 431, 434.xx, 436 for stroke; and 402.11, 402.91, 404.x1, 404.x3, 428.xx for CHF. We also evaluated death separately.

We used analysis of variance (ANOVA) and chi-square tests to evaluate differences in continuous and categorical characteristics, respectively, across PTH change groups. We used Cox proportional hazard modeling to evaluate the association between short-term PTH response (within 40 days) to cinacalcet and time to first composite event. Follow-up began at day 40 after first cinacalcet prescription and extended to the earliest of an outcome of interest or censoring (i.e., transplant, modality change, transfer from a unit of the large dialysis organization, or maximum follow-up of 1 year). Models were adjusted for baseline demographic characteristics, laboratory values, primary cause of ESRD, comorbid conditions, phosphate binder use, time to cinacalcet initiation, and hospital days. We conducted parallel analyses stratified by baseline PTH category (300-600 pg/mL vs. ≥ 600 pg/mL). All analyses were conducted using SAS 9.1. (SAS Institute, Cary, NC).

The Hennepin County Medical Center Institutional Review Board, Minneapolis, Minnesota, reviewed and approved this retrospective study. A waiver of informed consent was granted as risk to patients was minimal and the study would have been otherwise impracticable.

## Results

**Figure 1:**
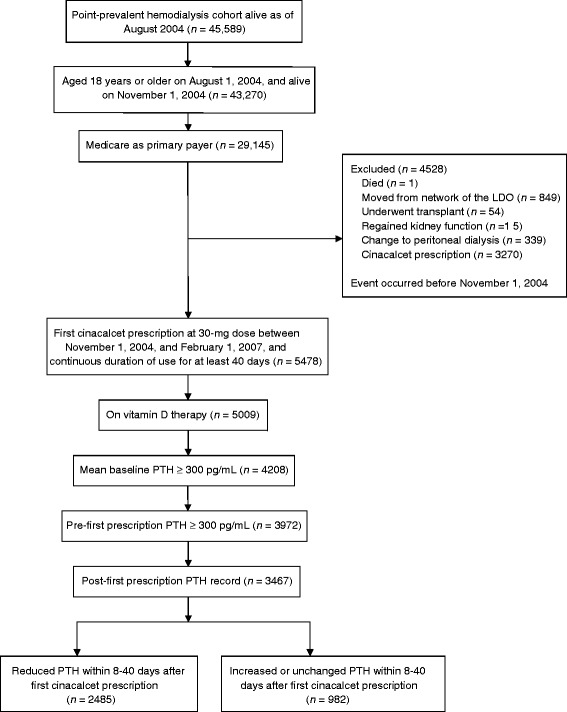
**Cohort creation, inclusion and exclusion criteria.** LDO, large dialysis organization; PTH, parathyroid hormone.

Of the 45,589 patients from the large dialysis organization who were available for analysis, 5478 (12%) met initial inclusion criteria, received a first cinacalcet prescription at a 30-mg dose between November 1, 2004, and February 1, 2007, and were on cinacalcet for at least 40 days. Of these, 3467 were receiving vitamin D before cinacalcet initiation, had mean baseline and pre-initiation PTH levels of 300 pg/mL or greater, and had a post-initiation PTH record within 8 to 40 days of first cinacalcet prescription (Figure 1). Only 17% of patients had more than one PTH record within that timeframe.

Of the 3467 included patients, 2485 (72%) experienced a decrease in PTH level within 8 to 40 days after cinacalcet initiation; the mean percentage change in PTH level was 43.4% (standard deviation, 25.8%; median, 42.0%). Patients whose PTH levels decreased were stratified into four groups based on the quartiles of percentage change from before to after cinacalcet initiation. A fifth group included 982 patients (28%) whose PTH levels increased or did not change; characteristics of each group are displayed in Table 1. Several characteristics differed between PTH response groups. Patients in quartile 4 (highest response), compared with patients in quartile 1 (lowest response), were older and more likely to be female, with lower PTH, slightly lower corrected calcium, and lower phosphorus before cinacalcet initiation. A higher proportion of quartile-4 patients had diabetes as ESRD cause, and ASHD, CHF, PVD, and diabetes as baseline comorbid conditions. Those relationships generally held true for patients with PTH greater than 600 pg/mL at baseline, but not for patients with PTH 300-600 pg/mL (see Additional file 1). Compared with the quartile groups, the group with no PTH reduction included a higher proportion of African Americans and lower proportions of patients with diabetes as ESRD cause or comorbid condition. Mean baseline PTH level was lowest for this group, and corrected baseline calcium and phosphorus levels were highest.

**Table 1:** **Baseline characteristics of patients in five PTH response groups**

	**PTH change group**					
**Patient characteristics**	**No PTH reduction**	**Quartile 1**	**Quartile 2**	**Quartile 3**	**Quartile 4**	***P***
*n*	982	621	621	621	622	
Mean (SD) percent change in PTH	+38.5 (43.9)	−11.2 (6.1)	−31.8 (5.8)	−52.2 (6.1)	−78.2 (9.2)	NA
Age, yrs.						
Mean (SD)	57.8 (15.0)	57.7 (14.7)	59.2 (14.4)	61.0 (14.1)	62.6 (13.4)	<0.0001
18-44	20.7	21.1	17.4	13.7	10.0	<0.0001
45-64	44.7	43.6	45.3	43.8	43.1	
65-74	20.7	22.1	23.8	25.3	28.1	
≥75	14.0	13.2	13.5	17.2	18.8	
Race						
African American	53.1	51.9	49.6	50.6	48.9	0.48
White	24.8	26.9	28.2	27.5	25.4	
Other	22.2	21.3	22.2	21.9	25.7	
Male sex	53.8	54.9	54.9	49.6	46.5	0.01
Dialysis duration, yrs., mean (SD)	6.0 (4.6)	5.8 (4.4)	5.8 (4.1)	5.4 (4.0)	5.8 (4.4)	0.3
BMI, kg/m^2^, mean (SD)	27.5 (7.2)	27.7 (6.9)	27.9 (7.0)	27.9 (7.2)	27.1 (6.3)	0.23
Intact PTH, pg/mL, pre-cinacalcet prescription						
Mean (SD)	687 (387)	798 (500)	756 (454)	739 (379)	740 (386)	
>300- ≤ 600	54.9	42.8	46.7	45.7	44.7	<0.0001
>600	45.1	57.2	53.3	54.3	55.3	
Corrected calcium, mg/dL						
Mean (SD)	9.83 (0.7)	9.70 (0.7)	9.70 (0.7)	9.72 (0.7)	9.64 (0.7)	
<9.0 mg/dL	11.0	14.7	11.1	10.6	13.7	<0.0001
≥9.0- ≤ 10.2	60.2	62.3	68.4	69.2	70.6	
>10.2	28.8	23.0	20.5	20.1	15.8	
Phosphorus, mg/dL						
Mean (SD)	6.4 (1.8)	6.4 (1.6)	6.1 (1.5)	5.9 (1.5)	5.9 (1.5)	<0.0001
<3.5 mg/dL	2.6	1.5	2.3	3.4	1.9	0.0002
≥3.5– ≤ 5.0	19.7	19.5	22.5	25.1	28.8	
>5.0	77.8	79.1	75.2	71.5	69.3	
Kt/V, mean (SD)	1.7 (0.4)	1.7 (0.4)	1.7 (0.5)	1.7 (0.4)	1.7 (0.4)	0.85
Primary cause of ESRD						
Diabetes	36.1	38.2	41.2	38.0	45.2	0.07
Hypertension	34.6	33.2	31.1	34.6	31.2	
Glomerulonephritis	13.2	11.9	13.5	11.0	10.6	
Other	16.1	16.8	14.2	16.4	13.0	
Hospital days						
0	60.6	65.4	64.3	60.4	59.8	0.53
1-2	8.4	7.3	7.1	9.5	7.1	
3-5	10.5	9.5	9.2	10.3	11.7	
>5	20.6	17.9	19.5	19.8	21.4	
Phosphate binder use at first cinacalcet prescription						
Calcium containing	41.0	40.6	39.6	40.4	44.1	0.56
Non-calcium containing	81.3	80.4	80.8	79.6	77.0	0.30
Combination	26.8	24.8	24.0	24.8	24.4	0.72
None	4.5	3.9	3.5	4.8	3.4	0.62
Comorbidity						
ASHD	26.0	24.2	27.5	32.4	28.8	0.01
CHF	26.3	25.0	25.4	28.2	28.6	0.49
CVA/TIA	9.2	8.9	8.4	9.7	9.5	0.94
PVD	24.5	23.2	24.6	27.9	28.9	0.09
Other cardiac disease	23.0	24.6	24.0	21.9	22.0	0.73
COPD	11.4	11.6	11.1	11.9	12.7	0.92
GI disease	5.5	4.2	5.2	6.6	6.6	0.29
Liver disease	2.4	1.8	1.3	2.7	2.4	0.38
Dysrhythmia	16.5	17.4	18.5	19.7	18.8	0.53
Cancer	3.6	5.6	4.8	6.3	4.3	0.11
Diabetes	47.9	50.9	53.0	53.1	55.7	0.03
Days from last PTH level to first cinacalcet prescription	15.4 (13.5)	14.2 (12.1)	13.1 (10.9)	14.3 (12.2)	14.7 (12.6)	0.01

Patients were grouped based on quartiles of PTH decrease from first cinacalcet prescription. Patients whose PTH levels increased or remained unchanged were assigned to “no PTH reduction” group.

Results are reported as percentage of patients or mean ± standard deviation. *P* values: We used analysis of variance (ANOVA) and chi-square tests to evaluate differences in continuous and categorical characteristics, respectively, across all PTH change groups.

ASHD, atherosclerotic heart disease; BMI, body mass index determined using height and weight; CHF, congestive heart failure; COPD, chronic obstructive pulmonary disease; CVA/TIA, cerebral vascular accident/transient ischemic attack; ESRD, end-stage renal disease; GI, gastrointestinal; PTH, parathyroid hormone; PVD, peripheral vascular disease; SD, standard deviation.

Mean follow-up time for the PTH change quartile groups and the group with no PTH reduction ranged from 0.72 years (quartile 3) to 0.76 years (quartile 2). Unadjusted intention-to-treat analyses showed a linear increase in the composite event rate (hospitalization for AMI, stroke, CHF, or death) from quartile 1 to quartile 4 when all patients were grouped together (Table 2). However, no specific patterns were seen when death was evaluated as the outcome or when patients were stratified by baseline PTH level (Table 2). Comparing PTH change quartiles 2, 3, and 4 with quartile 1 (lowest response) using Cox regression (unadjusted) showed no association between greater PTH reduction and either the composite outcome or death (Tables 3 and 4).

**Table 2:** **Unadjusted composite event and death rates by PTH change group at baseline with 1-year follow-up**

	**PTH response group**				
	**No PTH reduction**	**Quartile 1**	**Quartile 2**	**Quartile 3**	**Quartile 4**
**Composite event rates** ^*^					
All patients	49.6	46.4	48.5	50.0	50.5
PTH 300-600 pg/mL	47.2	42.7	48.5	44.1	48.1
PTH > 600 pg/mL	52.6	49.3	48.4	55.5	52.6
**Death rates**					
All patients	17.9	15.6	16.5	15.8	18.8
PTH 300-600 pg/mL	15.8	16.2	17.1	17.5	17.2
PTH > 600 pg/mL	20.5	15.1	16.1	14.3	20.0

Patients were grouped based on quartiles of PTH decrease from first cinacalcet prescription. Patients whose PTH levels increased or remained unchanged were assigned to “no PTH reduction” group.

Rates are expressed as events per 100 person-years.

^*^Composite events include death, or hospitalization for stroke, congestive heart failure, or acute myocardial infarction.

PTH, parathyroid hormone.

**Table 3:** **Unadjusted and adjusted hazards for composite event outcome by PTH change group (quartile) for all patients and stratified by baseline PTH**

**Composite event** ^*^				
**PTH change group**	**Unadjusted**		**Adjusted**	
**All patients**	HR (95% CI)	*P*	HR (95% CI)	*P*
No PTH reduction	1.07 (0.90-1.26)	0.5	1.11 (0.94-1.32)	0.2
Quartile 1	Referent		Referent	
Quartile 2	1.04 (0.87-1.26)	0.7	1.01 (0.84-1.22)	0.9
Quartile 3	1.07 (0.89-1.29)	0.5	0.98 (0.81-1.18)	0.8
Quartile 4	1.09 (0.91-1.31)	0.4	0.96 (0.79-1.16)	0.6
**Baseline PTH 300-600 pg/mL**				
No PTH reduction	1.10 (0.86-1.42)	0.5	1.14 (0.88-1.47)	0.3
Quartile 1	Referent		Referent	
Quartile 2	1.13 (0.86-1.50)	0.4	1.10 (0.83-1.46)	0.5
Quartile 3	1.03 (0.77-1.37)	0.8	0.91 (0.68-1.22)	0.5
Quartile 4	1.13 (0.85-1.49)	0.4	0.98 (0.73-1.31)	0.9
**Baseline PTH > 600 pg/mL**				
No PTH reduction	1.06 (0.85-1.34)	0.6	1.08 (0.86-1.37)	0.5
Quartile 1	Referent		Referent	
Quartile 2	0.98 (0.77-1.26)	0.9	0.96 (0.75-1.24)	0.8
Quartile 3	1.12 (0.88-1.42)	0.4	1.0 (0.78-1.28)	1.0
Quartile 4	1.06 (0.84-1.35)	0.6	0.93 (0.73-1.20)	0.6

Patients were grouped based on quartiles of PTH decrease from first cinacalcet prescription. Patients whose PTH levels increased or remained unchanged were assigned to “no PTH reduction” group.

^*^Composite events include death, or hospitalization for stroke, congestive heart failure, or acute myocardial infarction.

CI, confidence interval; HR, hazard ratio; PTH, parathyroid hormone.

**Table 4:** **Unadjusted and adjusted hazards for death by PTH change group (quartile) for all patients and stratified by baseline PTH**

**Death**				
**PTH change group**	**Unadjusted**		**Adjusted**	
**All patients**	HR (95% CI)	*P*	HR (95% CI)	*P*
No PTH reduction	1.15 (0.89-1.49)	0.3	1.23 (0.94-1.61)	0.1
Quartile 1	Referent		Referent	
Quartile 2	1.06 (0.79-1.42)	0.7	1.05 (0.78-1.41)	0.8
Quartile 3	1.01 (0.75-1.36)	0.9	0.95 (0.71-1.29)	0.7
Quartile 4	1.20 (0.91-1.60)	0.2	1.03 (0.77-1.39)	0.8
**Baseline PTH 300-600 pg/mL**				
No PTH reduction	0.98 (0.67-1.43)	0.9	1.05 (0.70-1.55)	0.8
Quartile 1	Referent		Referent	
Quartile 2	1.06 (0.69-1.62)	0.8	1.09 (0.70-1.69)	0.7
Quartile 3	1.08 (0.71-1.65)	0.7	0.98 (0.63-1.52)	0.9
Quartile 4	1.06 (0.69-1.63)	0.8	0.92 (0.59-1.44)	0.7
**Baseline PTH > 600 pg/mL**				
No PTH reduction	1.36 (0.95-1.94)	0.1	1.47 (1.02-2.13)	0.04
Quartile 1	Referent		Referent	
Quartile 2	1.06 (0.71-1.59)	0.8	1.04 (0.69-1.56)	0.9
Quartile 3	0.95 (0.63-1.43)	0.8	0.90 (0.59-1.37)	0.6
Quartile 4	1.32 (0.91-1.94)	0.2	1.16 (0.78-1.73)	0.5

Patients were grouped based on quartiles of PTH decrease from first cinacalcet prescription. Patients whose PTH levels increased or remained unchanged were assigned to “no PTH reduction” group.

CI, confidence interval; HR, hazard ratio; PTH, parathyroid hormone.

Likewise, after adjustment for multiple baseline characteristics and time to first cinacalcet prescription, no association was seen between short-term cinacalcet PTH change and risk of the composite event or death overall or in cohorts stratified by baseline PTH levels (Tables 3 and 4).

## Discussion

This study evaluated the association of short-term change in PTH levels and clinical outcomes over the year after first cinacalcet prescription. Our study results showed that greater percentage reductions in PTH levels in a short timeframe were not associated with a reduction in a composite outcome of death and cardiovascular events or death alone over 1 year. In addition, we found no association between short-term PTH change in models stratified by baseline PTH level or in models with death evaluated as an outcome.

Cinacalcet is a calcimimetic agent used in the management of SHPT in dialysis patients. In 2024, cinacalcet and other oral MBD medications are expected to be included in the monthly dialysis PPS bundled payment. In a capitated environment, it would be ideal if a surrogate marker could identify patients whose clinical outcomes would be more likely to improve with cinacalcet. Clinical studies have shown that PTH response to cinacalcet is rapid, with nadir PTH concentration occurring approximately 3 hours after dosing; PTH concentrations remain suppressed at 24 hours as compared with baseline [12]. Cinacalcet half-life is about 6 hours and the terminal half-life ranges from 30 to 40 hours. Steady state cinacalcet blood concentrations are reached within a week [11]. In an early clinical trial, all patients receiving cinacalcet doses 25 mg or higher exhibited decreased PTH concentrations following cinacalcet treatment, and concentrations appeared to level out after 5 to 7 days following a 50-mg daily dose [12]. Phase 3 clinical trials showed that the average reduction in PTH ranged from 40.5% to 43% in hemodialysis patients receiving daily doses of cinacalcet over 26 weeks when doses were titrated to achieve a mean intact PTH concentration of 250 pg/mL or less [1,3]. Based on evidence from these studies, and to reduce the chance of dose titration, we chose to evaluate PTH response to a 30-mg dose of cinacalcet within a relatively small window of 8 to 40 days after first cinacalcet prescription. To reduce the problem of misclassification over time between cinacalcet and non-cinacalcet groups, we chose to evaluate both PTH change and outcomes within relatively short time frames.

However, our study results did not support the hypothesis that short-term changes in PTH levels following cinacalcet initiation are associated with reduction in a composite of cardiovascular events and mortality in a 1-year follow-up. Our study findings could be related to cinacalcet discontinuation or nonadherence during the study timeframe. In a recent study using the same database as in this analysis, we showed that 30% of patients who initiated cinacalcet discontinued within 12 months [14]. However, we also showed that 51% of these patients reinitiated cinacalcet within 12 months of discontinuation. Gincherman and colleagues showed that the average refill rate for cinacalcet over the first 12 months was only 56%, and adherence decreased over time [15]. We did not measure the rates of discontinuation or re-initiation in this study, as we wished to determine if a relatively short-term response marker (change in PTH levels within 40 days of first cinacalcet prescription) could be used in real-life clinical practice to predict individual patients who would be more likely to experience improved cardiovascular and survival outcomes from cinacalcet. We assessed the PTH level drawn closest to 40 days to allow patients to develop daily dosing patterns. This PTH level would reflect a combination of cinacalcet’s PTH-lowering effect and current patient adherence. But as cinacalcet adherence appears to decrease over time [15], the potential for significant exposure misclassification cannot be ruled out.

Short-term changes in PTH following cinacalcet initiation may be modified by several factors, including non-adherence, timing of PTH levels in relation to the cinacalcet dose, and PTH variability. We used a dialysis prescription database to determine cinacalcet initiation, but we lacked information regarding whether or when patients actually filled or ingested their doses. Most dialysis patients are on a regular dialysis schedule, and laboratory values are consistently drawn before dialysis at the same time of day. However, as nadir PTH concentrations occur about 3 hours after a cinacalcet dose [12], changes in daily cinacalcet dosing patterns in relationship to the laboratory draw can affect the apparent PTH responsiveness in individual patients. The inherent variation in PTH levels may also have contributed to our null findings; Gardham and colleagues showed that dialysis patients have a higher biologic variability for PTH measurement than normal healthy volunteers [16]. Twenty-eight percent of patients in our study had increased or unchanged PTH levels after first cinacalcet prescription; this may have been due to true non-response, non-adherence, or PTH variability. In addition, the cinacalcet initiation date may have been inaccurate for some patients.

Finally, PTH changes over 40 days following cinacalcet initiation may not correlate to long-term PTH changes or to 1-year outcomes. Cinacalcet discontinuation is common [14,17], and even though many patients eventually reinitiate [14], patients may not have had enough cinacalcet exposure over 1 year to modify and sustain changes in chronic kidney disease MBD biochemical parameters sufficient to affect cardiovascular events and mortality.

The strengths of our study include a large patient population from one large dialysis organization that used only two clinical laboratories in Florida for PTH analysis. We evaluated PTH change in response to a standard 30-mg daily dose over a relatively short time period to reduce the chance that dose titration would occur. We also evaluated outcomes over a short time period (1 year) in cinacalcet users only to reduce the risk of misclassification from treatment discontinuation.

## Conclusions

In conclusion, our study results showed no relationship between short-term PTH change after first cinacalcet prescription and a combined endpoint of hospitalization for cardiovascular events or death over the following year. Thus, short-term changes in PTH after first cinacalcet prescription with a standard initial daily dose of 30 mg may not be a good surrogate for longer-term improvements in cardiovascular health or mortality. More evaluation is needed to identify surrogate markers. These evaluations should use databases with prescription fill information or electronic monitoring tools to assess patient adherence to cinacalcet to reduce the risk of misclassification bias.

## Additional file

Additional file 1: Table A1. Baseline characteristics of patients in five PTH change groups, baseline PTH 300-600 pg/mL. **Table A2.** Baseline characteristics of patients in five PTH change groups, baseline PTH > 600 pg/mL.

## Abbreviations

AMI: Acute myocardial infarction
ANOVA: Analysis of variance
ASHD: Atherosclerotic heart disease
BMI: Body mass index
CHF: Congestive heart failure
IV: Intravenous
Kt/V: Dialysis dose
MBD: Mineral and bone disorder
PPS: Prospective payment system
PTH: Parathyroid hormone
PVD: Peripheral vascular disease
SHPT: Secondary hyperparathyroidism
USRDS: United States Renal Data System
