# Supplementary material for: Parathyroid hormone change after cinacalcet initiation and one-year clinical outcome risk: a retrospective cohort study
Source: BMC Nephrol. 2015 Mar 31;16:41. doi: 10.1186/s12882-015-0030-8 (PMC4383071; doi:10.1186/s12882-015-0030-8)
Supplement: Additional file 1: Table A1. — Baseline characteristics of patients in five PTH change groups, baseline PTH 300-600 pg/mL. Table A2. Baseline characteristics of patients in five PTH change groups, baseline PTH > 600 pg/mL. [file 12882_2015_30_MOESM1_ESM.pdf]

Additional File 1

Table A1. Baseline characteristics of patients in five PTH change groups, baseline PTH 300-600 pg/mL

Patient Characteristics	PTH Change Group Quartile 1	Quartile 2	Quartile 3	Quartile 4	No PTH Reduction	*P*
n	266	290	284	278	539	
Mean (SD) percent change in PTH	-10.9 (6.0)	-31.7 (5.9)	-52.6 (6.1)	-77.5 (9.0)	+46.6 (40.4)	NA
Age, yrs.						
Mean (SD)	60.0 (14.5)	61.1 (14.1)	63.3 (13.6)	64.9 (13.2)	59.2 (15.0)	< 0.0001
18-44	17.7	14.1	9.9	7.2	17.1	0.001
45-64	40.6	42.4	40.5	40.3	44.9	
65-74	24.8	26.2	27.1	28.4	22.1	
≥ 75	16.9	17.2	22.5	24.1	16.0	
Race						
African American	49.3	42.8	45.4	43.2	51.6	0.03
White	29.7	35.5	32.4	27.7	25.8	
Other	21.1	21.7	22.2	29.1	22.6	
Male sex	55.3	55.9	48.2	48.2	54.7	0.01
Dialysis duration, yrs., mean (SD)	5.6 (4.3)	5.1 (3.9)	5.1 (4.2)	5.3 (3.9)	5.9 (4.6)	0.03
BMI, kg/m^2, mean (SD)	27.3 (6.5)	28.4 (7.5)	28.0 (7.2)	27.7 (6.7)	27.5 (7.0)	0.3
Intact PTH, pg/mL, pre-cinacalcet prescription, mean (SD)	455.0 (83.0)	454.0 (81.0)	454.0 (81.0)	457.0 (78.0)	447.0 (81.0)	0.40
Corrected calcium, mg/dL, mean (SD)	9.8 (0.7)	9.7 (0.7)	9.8 (0.6)	9.7 (0.7)	9.9 (0.7)	0.001
< 9.0	12.4	11.4	8.1	11.2	10.0	0.3
≥ 9.0-≤ 10.2	61.7	65.5	69.0	71.2	57.7	
> 10.2	25.9	23.1	22.9	17.6	32.3	
Phosphorus, mg/dL, mean (SD)	6.0 (1.5)	5.7 (1.3)	5.7 (1.4)	5.8 (1.6)	6.2 (1.7)	< 0.0001
< 3.5	1.9	3.5	3.5	2.5	2.8	0.3
≥ 3.5-≤ 5.0	28.6	26.6	29.6	30.6	23.0	
> 5.0	70.0	70.0	69.9	69.9	74.2	
Kt/V mean (SD)	1.7 (0.4)	1.7 (0.4)	1.7 (0.3)	1.7 (0.3)	1.7 (0.4)	0.8
Primary cause of ESRD						
Diabetes	40.2	43.1	40.1	46.8	38.0	0.2
Hypertension	34.2	30.3	38.0	31.7	34.1	
Glomerulonephritis	9.4	12.4	8.1	11.2	12.6	
Other	16.2	14.1	13.7	10.4	15.2	
Hospital days						
0	70.7	64.8	62.7	65.8	62.5	0.4
1-2	5.3	7.2	9.9	6.1	7.8	
3-5	8.7	8.3	11.3	8.3	10.0	
> 5	15.4	19.7	16.2	19.8	19.7	

Phosphate binder use at cinacalcet first prescription						
Calcium containing	39.5	40.7	39.4	45.7	41.0	0.6
Non-calcium containing	78.2	81.7	77.5	77.0	79.2	0.7
Combination	21.4	25.9	23.2	25.9	24.7	0.7
None	3.8	3.5	6.3	3.2	4.5	0.4
Comorbidity						
ASHD	25.6	30.3	34.9	29.5	26.0	0.06
CHF	25.9	25.9	28.2	27.7	25.2	0.9
CVA/TIA	7.1	6.6	7.8	9.4	10.0	0.4
PVD	23.3	25.2	28.9	28.4	25.1	0.5
Other cardiac disease	23.3	25.2	19.0	21.6	20.0	0.3
COPD	13.2	9.7	10.2	13.0	12.2	0.6
GI disease	2.6	4.8	6.3	5.8	5.0	0.3
Liver disease	1.5	2.1	2.5	1.1	1.9	0.8
Dysrhythmia	15.6	17.9	18.3	18.7	17.8	0.9
Cancer	7.1	4.5	6.0	4.3	4.1	0.3
Diabetes	53.4	55.5	53.9	58.6	51.0	0.3
Days from last PTH level to first cinacalcet prescription	13.8 (11.6)	12.8 (11.2)	13.6 (12.0)	14.9 (13.0)	15.3 (13.5)	0.05

Patients were grouped based on quartiles of PTH decrease from first cinacalcet prescription. Patients whose PTH levels increased or remained unchanged were assigned to “no PTH reduction” group.

Results are reported as percent of patients or mean ± SD. *P* values: We used analysis of variance (ANOVA) and chi-square tests to evaluate differences in continuous and categorical characteristics, respectively, across all PTH change groups.

ASHD, atherosclerotic heart disease; BMI, body mass index determined using height and weight; CHF, congestive heart failure; COPD, chronic obstructive pulmonary disease; CVA/TIA, cerebral vascular accident/transient ischemic attack; ESRD, end-stage renal disease; GI, gastrointestinal; PTH, parathyroid hormone; PVD, peripheral vascular disease; SD, standard deviation.

Table A2. Baseline characteristics of patients in five PTH change groups, baseline PTH > 600 pg/mL

	PTH Change Group					
Patient Characteristics	Quartile 1	Quartile2	Quartile 3	Quartile 4	No PTH Reduction	*P*
n	355	331	337	344	443	
Mean (SD) percent change in PTH	-11.4 (6.2)	-31.9 (5.8)	-51.9 (6.2)	-78.8 (9.3)	+28.7 (31.8)	NA
Age, yrs.						
Mean (SD)	55.9 (14.7)	57.6 (14.4)	59.0 (14.3)	60.7 (13.4)	56.1 (15.0)	< 0.0001
18-44	23.7	20.2	16.9	12.2	25.1	0.001
45-64	45.9	47.7	46.6	45.4	44.5	
65-74	20.0	21.8	23.7	27.9	19.0	
≥ 75	10.4	10.8	12.8	14.5	11.5	
Race						
African American	53.8	55.6	54.9	53.5	54.9	
White	24.8	21.8	23.4	23.6	23.6	
Other	21.4	22.7	21.7	23	23	
Male sex	54.7	54.1	50.7	45.1	52.6	
Dialysis duration, yrs., mean (SD)	5.8 (4.4)	6.4 (4.3)	5.6 (3.8)	6.1 (4.8)	6.2 (4.5)	
BMI, mean (SD)	28.0 (7.2)	27.5 (6.5)	27.9 (7.2)	26.7 (5.9)	27.6 (7.4)	
Intact PTH, pg/mL, pre-cinacalcet prescription, mean (SD)	1055.0 (528.0)	1021 (481.0)	979.0 (365.0)	969.0 (384.0)	981.0 (409.0)	0.045
Corrected calcium, mg/dL						
Mean (SD)	9.7 (0.7)	9.7 (0.7)	9.7 (0.7)	9.6 (0.7)	9.8 (0.7)	
< 9.0	16.3	10.9	12.8	15.7	12.2	
≥ 9.0-≤ 10.2	62.8	71.0	69.4	70.1	63.2	
> 10.2	20.0	18.1	17.8	14.2	24.6	
Phosphorus, mg/dL						
Mean (SD)	6.7 (1.6)	6.4 (1.5)	6.1 (1.6)	6.0 (1.5)	6.6 (1.8)	< 0.0001
< 3.5	1.1	1.2	3.3	1.5	2.3	
≥ 3.5-≤ 5.0	12.7	19.0	21.4	27.3	15.6	
> 5.0	86.2	79.8	75.4	71.2	82.2	
Kt/V mean (SD)	1.6 (0.5)	1.6 (0.5)	1.7 (0.4)	1.7 (0.4)	1.7 (0.5)	0.8
Primary cause of ESRD						
Diabetes	36.6	39.6	36.2	43.9	33.6	0.4
Hypertension	32.4	31.7	31.8	30.8	35.2	
Glomerulonephritis	13.8	14.5	13.4	10.2	14	
Other	17.2	14.2	18.7	15.1	17.2	
Hospital days						
0	61.4	63.8	58.5	54.9	58.2	0.5
1-2	8.7	7.0	9.2	7.9	9	
3-5	10.1	10.0	9.5	14.5	11.1	
> 5	19.7	19.3	22.9	22.7	21.7	
Phosphate binder use at first cinacalcet						

prescription						
Calcium containing	41.4	38.7	41.3	42.7	41.1	0.9
Non-calcium containing	82.0	80.1	81.3	77.0	83.8	0.2
Combination	27.3	22.4	26.1	23.3	29.4	0.2
None	3.9	3.6	3.6	3.5	4.5	0.9
Comorbidity						
ASHD	23.1	25.1	30.3	28.2	26.0	0.2
CHF	24.2	25.1	28.2	29.4	27.5	0.5
CVA/TIA	10.1	10.0	11.3	9.6	8.1	0.7
PVD	23.1	24.2	27.0	29.4	23.9	0.3
Other cardiac disease	25.6	23.0	24.3	22.4	26.6	0.6
COPD	10.4	12.4	13.4	12.5	10.4	0.6
GI disease	5.4	5.5	6.8	7.3	6.1	0.8
Liver disease	2.0	0.6	3.0	3.5	3.2	0.1
Dysrhythmia	18.6	19.0	20.8	18.9	14.9	0.3
Cancer	4.5	5.1	6.5	4.4	2.9	0.2
Diabetes	49.0	50.8	52.5	53.5	44	0.06
Days from last PTH level to first cinacalcet prescription	14.5 (12.4)	13.4 (10.6)	14.8 (12.4)	14.5 (12.3)	15.5 (13.4)	0.2

Patients were grouped based on quartiles of PTH decrease from first cinacalcet prescription. Patients whose PTH levels increased or remained unchanged were assigned to “no PTH reduction” group.

Results are reported as percent of patients or mean ± SD. *P* values: We used analysis of variance (ANOVA) and chi-square tests to evaluate differences in continuous and categorical characteristics, respectively, across all PTH change groups.

ASHD, atherosclerotic heart disease; BMI, body mass index determined using height and weight; CHF, congestive heart failure; COPD, chronic obstructive pulmonary disease; CVA/TIA, cerebral vascular accident/transient ischemic attack; ESRD, end-stage renal disease; GI, gastrointestinal; PTH, parathyroid hormone; PVD, peripheral vascular disease; SD, standard deviation.
